# The neuroprotective effects of Lutongkeli in traumatic brain injury rats by anti-apoptosis mechanism

**DOI:** 10.1590/acb370603

**Published:** 2022-09-19

**Authors:** Qiu-Xia Xiao, Lu-Lu Xue, Zhang-Yu Su, Jin Huang, Ji-Lin Chen, Liu-Lin Xiong, Ting-Hua Wang

**Affiliations:** 1 MD. Kunming Medical University – Institute of Neuroscience – Animal Zoology Department – Kunming, China.; 2 PhD. Sichuan University – State Key Laboratory of Biotherapy – Chengdu, China.; 3 BS. Southwest Medical University – Department of Anesthesiology – Luzhou, China.; 4 PhD. Kunming Medical University – Affiliated Hospital – Department of Neurosurgery – Kunming, China.; 5 BS. Kunming Medical University – Institute of Neuroscience – Animal Zoology Department – Kunming, China.; 6 PhD, Professor. Kunming Medical University – Institute of Neuroscience – Animal Zoology Department – Kunming, China.

**Keywords:** Brain Injuries, Traumatic, Lutongkeli, Network Pharmacology, Apoptosis, Neuroprotection

## Abstract

**Purpose:**

To explore the neuroprotective effects of Lutongkeli (LTKL) in traumatic brain injury (TBI) and detect the related mechanism.

**Methods:**

TBI model was established with LTKL administration (2 and 4 g/kg/d, p.o.). Motor function of rats was examined by Rotarod test. Nissl staining was used to show neuron morphology. Furthermore, the disease-medicine common targets were obtained with the network pharmacology and analyzed with Kyoto Encyclopedia of Genes and Genomes. Lastly, the predicted targets were validated by real-time polymerase chain reaction.

**Results:**

After LTKL administration, neural behavior was significantly improved, and the number of spared neurons in brain was largely increased. Moreover, 68 bioactive compounds were identified, corresponding to 148 LTKL targets; 2,855 genes were closely associated with TBI, of which 87 overlapped with the LTKL targets and were considered to be therapeutically relevant. Functional enrichment analysis suggested LTKL exerted its pharmacological effects in TBI by modulating multiple pathways including apoptosis, inflammation, etc. Lastly, we found LTKL administration could increase the mRNA level of Bcl-2 and decrease the expression of Bax and caspase-3.

**Conclusions:**

This study reported the neuroprotective effect of LTKL against TBI is accompanied with anti-apoptosis mechanism, which provides a scientific explanation for the clinical application of LTKL in the treatment of TBI.

## Introduction

Traumatic brain injury (TBI) is caused by the external violence to the head, which brings neurological abnormalities and even death^1^. With the rapid development of modern society, brain trauma has increasingly become a high incidence of disease^2^, and it is extensive accounted as a serious challenge to public health^3^. At present, drug therapy is one of the most important treatment methods including inflammatory inhibitors, neurotrophic factors, hormones, and oxygen free radical scavengers. Because of the complex pathogenesis of TBI, these methods have certain effect^4^^,^ 5. Thus, they prompt us to exploit the available medicine for the remedy of TBI.

Traditional Chinese medicine (TCM), extracted from the plant, animal, mineral, and processed products, has been applied in the prevention and treatment of diseases for a long time^6^. Because of its medical efficacy and therapeutic effectiveness, it is regarded as an important method of disease treatment^7^. Accumulated clinical data and basic experiments have showed that TCM has the specific therapeutic effects for the treatment of brain trauma^8^^,^ 9. However, due to the complex composition and unclear pharmacological mechanism, TCM has been hindered from its widespread use in clinical healthcare.

Network pharmacology is a new subject based on the theory of system biology, which analyses the network of biological system and selects specific signal nodes for multi-targets drug molecular design. Network pharmacology emphasizes the multi-channels regulation of signal pathway, improves the therapeutic effect of drugs, reduces adverse drug reaction^10^. Recently, network pharmacology has been gradually applied to the development and research domain of TCM. The combination of network pharmacology and TCM compound has unique advantage in the research of active drugs in TCM compounds. It is of great significance to predict and analyze the components and explain the molecular mechanism of compound prescription^11^. The research of network pharmacology can reveal the synergistic effect of traditional medicine prescription by acting on multiple targets and pathways at different levels, which bridges the gap between traditional medicine and modern medicine, greatly promotes the research on the synergistic effect of TCM compound prescription, and contributes to the development of medicine^12^^,^ 13.

Apoptosis, as a critical event, is the characteristic feature of the primary damage after TBI. Bcl-2, caspase3 and Bax, members of apoptosis genes, play an important role in the process of TBI^14^. Overexpression of Bcl-2 can effectively inhibit apoptosis induced by many factors, and Bax can promote apoptosis and cause mitochondria to release cytochrome C and caspase9 and, thus, promote cell apoptosis, while caspase3 is in the form of inactive proteinase under physiological condition. Whereas after activation, it can lyse the inherent and protective enzymes in cells, which induce the morphological and biochemical characteristics of cell apoptosis^15^. Therefore, how to alleviate cell apoptosis under drug administration is very important for the recovery of TBI patients.

Lutongkeli (LTKL), a TCM developed by Southwest Medical University, has the advantages of dispelling wind and dehumidifying, clearing collateral and relieving pain, which has been used for vascular headache and nerve headache. Therefore, this study was designated to detect the effect of LTKL for the TBI treatment, and explain the underlying mechanism, especially the role of anti-apoptosis reaction after TBI.

## Methods

### Animals and maintenance

Adult fit male Sprague-Dawley rats (210–240 g), housed under warm and 12 h light/dark cycle conditions with sufficient water and chow in Animal Center of Kunming Medical University, were used in the experiment. All procedures have been permitted by the Institutional Medical Experimental Animal Care Committee of Kunming Medical University, China (No. kmmu2021-759). Before the experiment, all animals were optionally separated into four groups: sham group, control group (NS), LTKL-medium dose group (LTM, 2 g/kg/d, p.o.) and LTKL-high dose group (LTH, 4 g/kg/d, p.o.) (N = 10 each group). All experimental protocols were complied with guidelines of the National Institutes of Health. Grouping and intervention of animals are shown in Table 1.

**Table 1:** The grouping and intervention of animals.

Groups	N	Intervention
Sham	10	Rats were subjected to the same process without TBI
NS	10	TBI + 2 mL of normal saline
LTM	10	TBI + 2 mL of LTKL (2 g/kg/d, p.o.)
LTH	10	TBI + 2 mL of LTKL (4 g/kg/d, p.o.)

NS: control group; LTM: Lutongkeli-medium dose group; LTH: Lutongkeli-high dose group; TBI: traumatic brain injury.

### Traumatic brain injury model

Rats TBI model was established by the modified Feeney’s method^16^. Later, the rats were given intraperitoneal anesthesia with pentobarbital sodium (30 mg/kg) and placed on a stereotactic frame^17^^,^ 18. After shaving and sterilization, a 1.5-cm midline scalp incision was established to display the right skull. Then, a 5-mm^2^ skull window was acquired by the dental drill, conforming to the designated coordinates as 0.2 cm after the coronal suture and 0.15 cm beside the sagittal suture. Next, a substance weighing 50 g was dropped from 25-cm height along a standing iron bar and directly affected on the right parietal brain tissue. The sham groups were subjected to the same process without the weight blow. Once the brain contusion was finished, the scalp was covered by bony wax, and the skin was sutured.

### Drug administration

LTKL is composed of *Notopterygium root*, *Ligusticum*, *Schizonepeta*, *Ligusticum wallichii*, *Scorpion*, *Fructus viticis*, *Pueraria*, etc., in a ratio of 3:4:2:4:1:2:4, which was extracted by ethanol to make flow extract, medicinal residue and other portions decocted to make clear paste, with dextrin as auxiliary material granulation. It was prepared and provided by the preparation room of the Hospital of Traditional Chinese Medicine affiliated to Southwest Medical University. The specification is 10 g/bag, and the production batch number is 20161009. Ten g of granules is equivalent to 17 g of crude drug, which prepared as a gavage solution with normal saline^19^. A dose of 4 and 2 g/kg/d from LTKL was calculated for the treatment of experimental rats. The concentration of drugs in the high-dose and medium-dose groups of rats was formed according to the standard volume of 2 mL, and 2 mL of saline was giving to control group. The doses of drugs included light, moderate and high that have been pre-experiment in the lab (data was not shown), so we chose high-dose and medium-dose in this study. Drugs were given by intragastric administration for 28 consecutive days in treatment groups, and the frequency is one time per day in the morning. The control group was subjected to saline treatment.

### Motor behavioral assessment-rotarod test

The experiment operation was carried out according to the previously mentioned way with little modulation^20^. Rats were put on a spinning girded, and the velocity of rotation was expedited from 10 to 30 rpm within 180 s. The experimental data was acquired as the average value of the three measurements.

### Nissl staining

Paraffin-embedded brain tissues were sectioned, deparaffinized, and then stained with 30 μL of 1% cresyl violet solution (Solarbio, Beijing, China) in a wet incubator for 9 min. After rinsed with distilled water, Nissl differentiation solution was added onto the sections for 2 min. Whereafter, 95% ethanol was added to differentiate swiftly until Nissl bodies were purple and other tissues were colorless. Lastly, specimens were dehydrated by absolute ethyl alcohol, transparentized by xylene and sealed by neutral gum. Images of the damaged cortex was obtained using a light microscope (Leica). Only complete neurons were counted in the Nissl-stained sections. Four sections were randomly selected from each rat, and five fields were randomly selected from each section to count the number of total neurons and dark neuron with ImageJ^21^. The percentage of neuron survival was calculated by Eq. 1:


Percentage of neuron survival = (number of total neurons - number of dark neurons) / number of total neurons (1)

### Prediction of target genes of traumatic brain injury

Disease-related target acquisition TBI disease-related genes were collected by using “traumatic brain injury” as the keyword from GeneCards database (https://www.genecards.org/). The collected disease targets were processed, and the redundant duplicated targets were deleted to obtain the relevant targets of TBI.

### Data mining of targets for Lutongkeli

The chemical compositions and targets of LTKL were obtained by searching the chemical composition information of LTKL with TCM Systems Pharmacology Database and Analysis Platform database (TCMSP) (http://tcmspw.com/tcmsp.php)^22^.The screening criteria for active ingredients were oral bioavailability (OB) ? 30% and drug-likeness (DL) ? 0.18^23^. With the help of Integrative Pharmacology-based Research Platform of TCM (http://www.tcmip.cn/)^24^, BATMAN platform (http://bionet.ncpsb.org/batman-tcm/)^25^ searched for the chemical components of which not included in TCMSP, and then collected the potential protein targets of the active components one by one. Finally, the protein name was standardized through the Uniprot database (https://www.uniprot.org/), and *Homo sapiens* was selected as the species, which was the potential target of the drug.

### The construction of disease-medicine-ingredients-target network

Through Venn Diagram online tool (http://bioinformatics.psb.ugent.be/webtools/Venn/), the common target genes of LTKL and TBI were acquired. In order to further understand the underlying mechanism, the Cytoscape 3.7.1 was employed to construct the network of LTKL bioactive ingredients and common targets.

### The analysis of Kyoto Encyclopedia of Genes and Genomes pathway

In this study, Kyoto Encyclopedia of Genes and Genomes (KEGG) pathway analysis https://www.kegg.jp/) were performed using bioconductor clusterProfiler, an R package was used for enrichment analysis of gene clusters^26^. Then, the KEGG pathways were ranked according to the p-value and gene count. The network of top 10 signaling pathways–correlative targeted gene were constructed, and the mechanism of LTKL against TBI was discussed.

### Real-time polymerase chain reaction verification

The peripheral cortex from different groups was collected respectively. The total RNA was extracted with Trizol reagents (Invitrogen Life Technologies, United States), then they were reversed transcripted as cDNA. Reaction for reverse transcription to cDNA was performed on the basis of the instruction of cDNA Synthesis Kit (Thermo Fisher Scientific). Next, quantitative real-time polymerase chain reaction (RT-PCR) was employed to evaluate the change of apoptosis-associated molecules, and the primer sequences information was as following:

Caspase3: F, 5-AACGAACGGACCTGTGG-3; R, 5-GGGTGCGGTAGAGTAAGC-3; Bax: F, 5-TGGAGCTGCAGAGGATGATT-3, R, 5-CAGGGCCTTGAGCACCAGTT-3; Bcl-2: F, 5-GAGGATTGTGGCCTTCTTTG-3, R, 5-GTTCCACAAAGGCATCCCAG-3; ß-actin: F, 5-GTAAAGACCTCTATGCCAACA-3; R: 5-GGACTCATCGTACTCCTGCT-3.

The amplified reaction was executed in a DNA thermal cycler (ABI 7300), and the amplified curve was acquired. Then, standard ^Δ^Ct value was calculated with β-actin as internal reference. The PCR amplification was following the next standard procedure: one cycle of 94 °C for 5 min; 35 cycles of 94 °C for 60 s; annealing for 60 s and 72 °C for 60 s. The relative expression of every gene was evaluated by the 2^-ΔΔ^Ct method.

### Statistical analysis

All values in the experiment are expressed as the mean ± standard deviation. Statistical analyses were executed using Prism 6 (Graphpad). One-way analysis of variance (ANOVA) was performed to detect the difference among all groups. The p-value < 0.05 was considered as statistically significance.

## Results

### Lutongkeli improves motor behavior in traumatic brain injury rats

**Figure 1:**
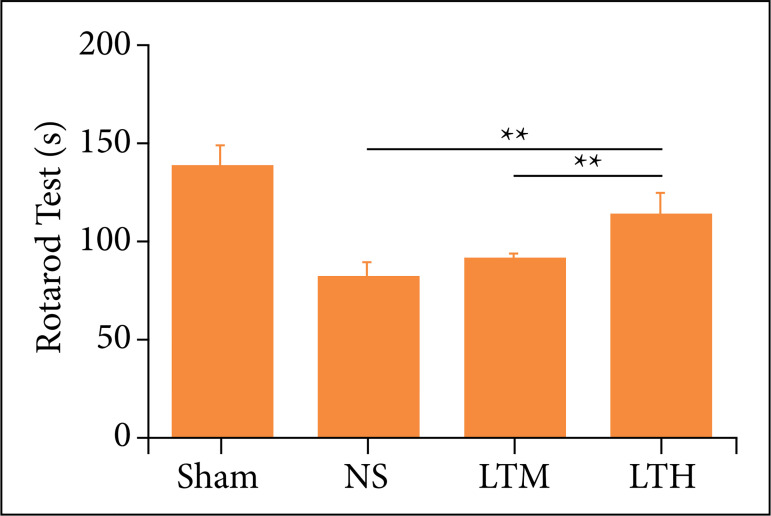
Lutongkeli improved motor function of traumatic brain injury rats. The results of rotarod test were presented at 14 days after the operation. Data were presented as the mean ± standard deviation, **(a)** The images of neurons after Nissl staining, scale bar=50μm. **(b)** The percentage of neuron suvival after LTH treatment.

The rotarod test showed that the performance of the LTKL treatment group was better than the control group, and the result of the high-dose group was the best (p < 0.05), which showed that the effect of LTKL was concentration-dependent, and the high-dose LTKL exhibited a positive effect, so as that a high-dose LTKL was used for the later experiment (Fig. 1).

** p < 0.01; NS: normal saline was giving to control group; LTM: Lutongkeli medium-dosegroup (2 g/kg/d, p.o.); LTH: Lutongkeli high-dose group (4 g/kg/d, p.o.).

### The number of spared neurons in Lutongkeli treatment

**Figure 2:**
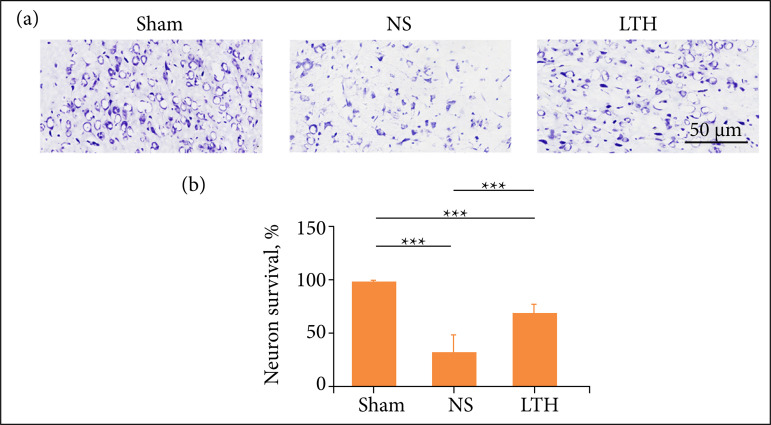
Lutongkeli treatment increases the survival rate of the nerve cell.Nissl staining was employed to check the neuronal degeneration, N = 10.

^***^p < 0.001; NS: normal saline was giving to control group; LTH: Lutongkeli high-dose group (4 g/kg/d, p.o.).

At day 1, the results of Nissl staining in each group were compared. The cortical nerve cells in sham group were clear blue and purple, and the nucleus was blue. The number of neurons was orderly and relatively large, with complete cell structure. Compared with the sham group, the NS group had relatively shallow staining, few neurons, most of them were in the state of apoptosis, and the survival rate was lower with statistically significant difference. The number of nerve cells in LTH group was less than that in sham group, and more than that in NS group. The difference was statistically significant (Fig. 2).

### Target prediction of Lutongkeli by bioinformatics

We searched herbs name in TCMSP database, and a total of 144, 159, 185, 82, 18 and 189 chemical ingredients of the six herbal medicines in LTKL formula were obtained from TCMSP database. Because no result was found about *Scorpion* in TCMSP, we further used BATMAN-TCM to search chemical ingredients with the keyword “QUAN XIE” and acquired seven chemical ingredients. Finally, according to the criteria of OB ≥ 30% and DL ≥ 0.18, 27, 11, 15, one, four, seven, and three active compounds were identified from seven herbal medicines, respectively (Table 2). Deleted replicated active compounds and target genes, 148 target genes in seven herbal medicines were obtained (Supplementary Table 1). Afterwards, we used the keyword “traumatic brain injury” in genecard to screen the disease-related genes and obtained a total of 2,855 (Supplementary Table 2).

**Table 2:** The compositions of Lutongkeli prescription.

Herb name	Compound	Active compound
*Viticis fructus*	144	27
*Schizonepetae herba*	159	11
*Notopterygii rhizoma et radix*	185	15
*Ligustici rhizoma et radix*	82	1
*Radix puerariae*	18	4
*Chuanxiong rhizoma*	189	7
*Scorpion*	7	3

### Construction of disease-medicine-active ingredients-target genes network

**Figure 3:**
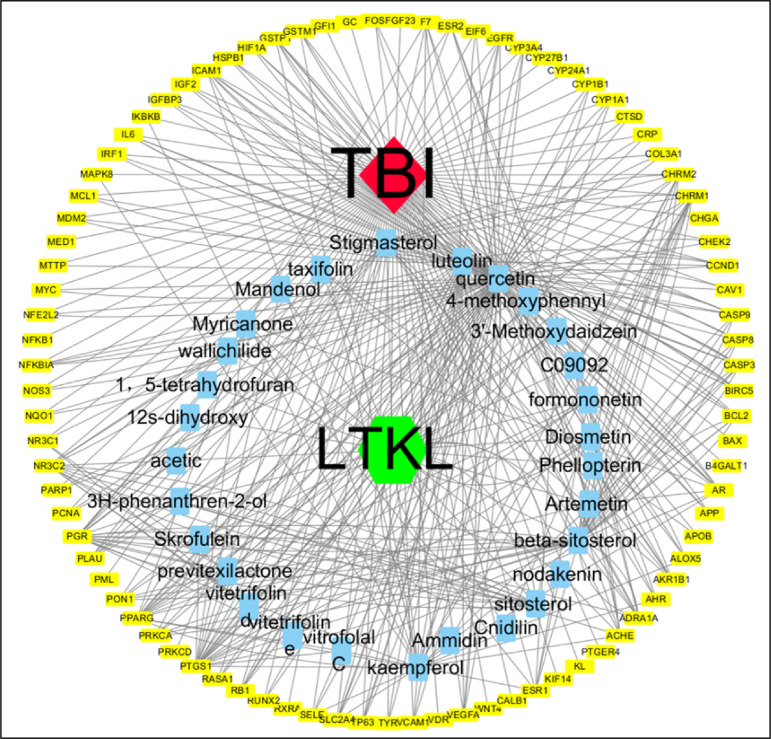
Disease-formula-active compounds-target network for traumatic brain injury. The red, green, blue, and yellow nodes represent the disease, herbs, active compounds and targets, respectively. The edges represent the interactions among them.

By taking the intersection of drug and disease targets, we acquired the total of 87 common genes between disease-related genes and drug-target genes (Supplementary Table 3). A network of LTKL ingredients-disease common target genes, composed of 122 nodes (one disease, one formula, 33 active compounds, and 87 targets), was established (Fig. 3).

### Kyoto Encyclopedia of Genes and Genomes enrichment analysis

**Figure 4:**
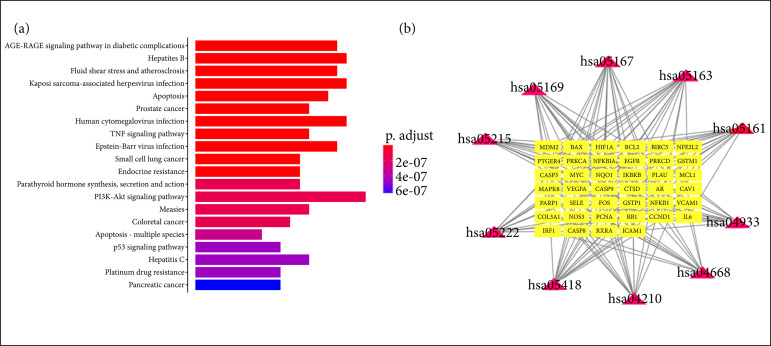
Kyoto Encyclopedia of Genes and Genomes enrichment analysis of common target gene. **(a)** The top 20signal pathway. **(b)** The top 10 target-pathway network for Lutongkeli. The red and yellow nodes representthe pathway and targets, respectively, and the edges represent the interactions among them.

In the KEGG signal pathway analysis, 111 relevant signal pathways were identified (Supplementary Table 4). The leading pathways of enrichment are mainly AGE-RAGE signaling pathway in diabetic complications, hepatitis B, fluid shear stress and atherosclerosis, Kaposi sarcoma-associated herpesvirus infection, apoptosis, prostate cancer, human cytomegalovirus infection, tumor necrosis factor (TNF) signaling pathway, etc. (Fig. 4a). The apoptosis signaling pathway rank by the front (p-value = 5.56E-1), and the participated genes are BAX, BCL2, BIRC5, CASP3, CASP8, CASP9, CTSD, FOS, IKBKB, MAPK8, MCL1, NFKB1, NFKBIA, PARP1. Furthermore, the first 10 pathways and the genes involved in these pathways are shown in Fig. 4b.

### Lutongkeli alleviates the apoptotic level

**Figure 5:**
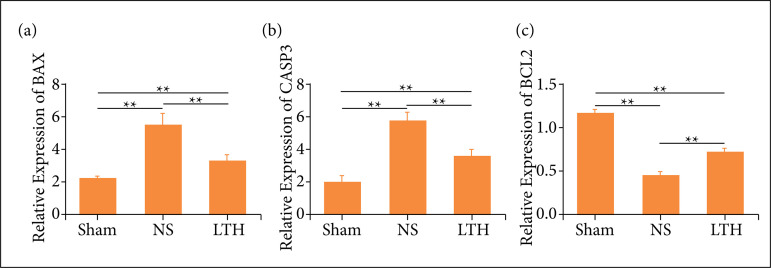
Lutongkeli treatment reduced the level of apoptotic cytokine and increased the level of anti-apoptotic cytokine. (**a, b, c**) Real-time polymerase chain reaction was employed to check the mRNA level of Bax, Caspase3and Bcl-2 at one-day post operation Data were presented as the mean ± standard deviation, N = 10.

^**^p < 0.01; NS: normal saline was giving to control group; LTH: Lutongkeli high-dose group (4 g/kg/d, p.o.). CASP3: Caspase3

The therapeutic effects of LTKL against nerve cells apoptosis caused by TBI were checked by RT-PCR. The level of the pro-apoptotic molecules, Bax and caspase3, were upregulated after TBI, compared with that of the sham group, while the mRNA level of the anti-apoptotic molecules, Bcl-2, was reduced compared with the sham group. Importantly, the level of Bax, caspase3, and Bcl-2 has been overturned in the TBI induced rats subjected to LTKL (Fig. 5).

## Discussion

In the present study, after LTKL administration, we found that it was effective for motor functional improvement in TBI rats. Moreover, LTKL increased the number of spared neurons in the injured brain tissue.

The network pharmacology approach was used to acquire 87 target genes, and functional enrichment analysis suggested that LTKL exerted its pharmacological effects in TBI by modulating multiple pathways, including apoptosis, and inflammation. Importantly, LTKL exerted a protection effect by adjusting apoptosis-related factors. These important findings confirmed that the administration of LTKL could improve athletic ability, and the molecular mechanism is associated with upregulating the expression of Bcl-2, reducing the level of Bax and caspase3.

### Lutongkeli-treated rats exhibited a positive motor improvement

Previous animal toxicological studies of LTKL did not find toxic and side effects and detected no LD50 at the maximum dose of acute toxicity experiment in rats. The maximum dose of rats measured in the experiment of 72 g/kg is equivalent to 144 times the human daily dose, indicating the safety of the drug. In this study, we found rats in LTKL-treated groups significantly increased the motor ability in rotarod tests at 14 days. At present, Chinese medicine has been widely applied in the treatment of clinical diseases^27^. LTKL has been applied in clinical therapy and presents a benefit effect for the health care. However, it is unclear whether LTKL has a neuroprotective effect in the treatment of TBI. In this experiment, we confirmed the effective role of LTKL in the motor functional improvement after TBI by using rotarod test, which provided an understanding for the usage of LTKL in clinic.

### Lutongkeli-treatment rats increased the spared neurons in traumatic brain injury rats

In morphological level, there is no evidence to know the effect of LTKL, except our report from this experiment. According to the literature, several TCMs were used to improve morphology, such as reducing neuronal apoptosis or survival of neurons^28^^,^ 29. These indicated that Chinese medicine is effective for the disease treatment, and this study contributes to novel finding in which LTKL remedy can protect neuron in the cortex from injury and this may improve the last neurological recovery.

### Network pharmacology analysis of LTKL

Our study showed that LTKL can improve the neurological behavior in rats with TBI. However, the sophisticated mechanisms of the above improvement were unknown. Therefore, we predicted the targets of LTKL via a systematic bioinformatics method that included TCMSP, TCMID, BATMAN-TCM and KEGG. These optimal systematic and comprehensive databases for pharmacology analyses identified the key pathway of LTKL to be apoptosis. After combining with previous results and the network analysis, apoptosis related factor (Bax, bcl-2 and caspase3) was chosen for further validation in the common genes. A large amount of evidence shows that the network pharmacology exerts a crucial role in the treatment of the nervous system, which provides an important basis for mechanism study^30^^,^ 31.

### Lutongkeli-therapy contributes to anti-apoptosis in traumatic brain injury rats

We also investigated the mechanism of LTKL on TBI. There are lower expression of Bax and caspase3 and higher expression Bcl-2 in LTKL treatment. The activity of caspase3 is involved in the loss of brain tissue after brain trauma and plays a crucial part in determining the activation of downstream events in cells^32^. Many studies have shown that apoptosis is closely related to brain trauma, and the apoptosis of nerve cells caused by brain trauma is an important form of cell death secondary to brain injury^33^^,^ 34. Some studies have detected the brain tissues of 18 patients with an acute craniocerebral injury who survived 5 to 10 h after injury and found that apoptotic nerve cells peaked at 24-28 h^35^. It was found that the level of Bax raised, and the expression of Bcl-2 reduced at 4 h after injury in the study on the chronology of nerve cell apoptosis after brain injury^36^. Calcium-activated protease and caspase3 are activated after craniocerebral injury, which is accompanied by cell necrosis and apoptosis^37^. Our study showed that LTKL plays a vital role in anti-neurotrauma, and the mechanism is associated with apoptosis.

## Conclusions

We demonstrated LTKL can improve the motor behavior in TBI injured rats, and its neuroprotective effect may be partly associated with morphological improvement and molecular regulation especially in anti-apoptosis reaction. These important findings would contribute to illuminate the neuroprotective effect of LTKL in TBI insult and explain the related anti-apoptosis mechanism, which is useful for the application of LTKL in the treatment of neurotrauma in future clinic practice.
